# The Influence of Antismoking Television Advertisements on Cessation by Race/Ethnicity, Socioeconomic Status, and Mental Health Status

**DOI:** 10.1371/journal.pone.0102943

**Published:** 2014-07-17

**Authors:** James M. Nonnemaker, Jane A. Allen, Kevin C. Davis, Kian Kamyab, Jennifer C. Duke, Matthew C. Farrelly

**Affiliations:** RTI International, Research Triangle Park, North Carolina, United States of America; Old Dominion University, United States of America

## Abstract

Disparities in tobacco use and smoking cessation by race/ethnicity, education, income, and mental health status remain despite recent successes in reducing tobacco use. It is unclear to what extent media campaigns promote cessation within these population groups. This study aims to (1) assess whether exposure to antitobacco advertising is associated with making a quit attempt within a number of population subgroups, and (2) determine whether advertisement type differentialy affects cessation behavior across subgroups. We used data from the New York Adult Tobacco Survey (NY-ATS), a cross-sectional, random-digit-dial telephone survey of adults aged 18 or older in New York State conducted quarterly from 2003 through 2011 (*N* = 53,706). The sample for this study consists of 9,408 current smokers from the total NY-ATS sample. Regression methods were used to examine the effect of New York State’s antismoking advertising, overall and by advertisement type (graphic and/or emotional), on making a quit attempt in the past 12 months. Exposure to antismoking advertising was measured in two ways: gross rating points (a measure of potential exposure) and self-reported confirmed recall of advertisements. This study yields three important findings. First, antismoking advertising promotes quit attempts among racial/ethnic minority smokers and smokers of lower education and income. Second, advertising effectiveness is attributable in part to advertisements with strong graphic imagery or negative emotion. Third, smokers with poor mental health do not appear to benefit from exposure to antismoking advertising of any type. This study contributes to the evidence about how cessation media campaigns can be used most effectively to increase quit attempts within vulnerable subgroups. In particular, it suggests that a general campaign can promote cessation among a range of sociodemographic groups. More research is needed to understand what message strategies might work for those with poor mental health.

## Background

Despite recent successes in reducing tobacco use, disparities in tobacco use and smoking cessation remain by race/ethnicity, education, income, and mental health status. National data sources show stark differences in smoking by race/ethnicity, with American Indian/Alaska Natives smoking at the highest rate, followed by those who identify with multiple racial/ethnic groups [1], [2]. Smoking prevalence is the same among black and white adults and lower among Hispanic adults [1], [2]. However, health care providers are less likely to talk with black and Hispanic smokers and advise them to quit [3]–[5], and they are less likely to use effective cessation aids [3], [6], [7]. Nevertheless, Hispanics and individuals who identify with more than one racial/ethnic group are more likely to have made a quit attempt than white smokers [8], and studies show no differences in quit success by race/ethnicity [9]. Smoking prevalence is markedly higher among those with less education and income [1], [10], [2] and poor mental health [11]–[12] than among those with more resources and better health. Education and income are also strongly negatively associated with successful quitting [4], [13].

Media campaigns can promote the use of cessation services (e.g., calls to a quitline [14]) and directly promote cessation [15]–[17]. A key question is whether media campaigns can also increase cessation within vulnerable population groups. Unfortunately, smoking cessation campaigns have not been routinely evaluated by race/ethnicity or socioeconomic status (SES), and those that have yield mixed results in terms of effectiveness [18]–[23]. A review by Bala et al. [19] found that California’s general population campaign produced smoking declines across all racial/ethnic groups (although declines were somewhat smaller among black women compared with white and Hispanic women) and among women who had not completed high school. Results for the Massachusetts state campaign have also been somewhat mixed. According to the review by Bala et al. [19], this campaign was only effective for men. However, a study by Durkin et al. [20] found the campaign had a significant effect on quitting among adults in general. Further, a review by Niederdeppe et al. [21] concluded that, of 18 campaigns designed for a general audience, nine were less effective, six were equally effective, and three were more effective among lower SES individuals compared with higher SES individuals.

Likewise, the evidence for effectiveness of campaigns specifically designed for racial/ethnic minority or low SES smokers is mixed. Niederdeppe et al. [21]–[22] found that, of 13 campaigns designed to influence low SES individuals, eight generated mixed or inconclusive results, and five were less effective among low SES individuals. More recently, a cessation media campaign designed to reach lower income, blue-collar smokers of diverse race/ethnicity was associated with increases in cessation-related cognitions among Hispanics, quit attempts among non-Hispanic blacks, and both cognitions and quit attempts among smokers with less than a high school education [23].

Evidence suggests that advertisement type may contribute to differential campaign effects by population subgroups. Niederdeppe et al. [24] found that smokers with low levels of education and income were more likely to recall, and to perceive as more effective, advertisements that used graphic imagery or personal testimonials to convey why to quit information compared with advertisements that did not use graphic imagery or strong emotional content and focused on how to quit.

Biener et al. [25] showed that advertising that elicits strong negative emotion resonates with smokers. A longitudinal study by Durkin et al. [20] found that advertising that elicits strong negative emotion can increase cessation rates in lower SES populations. Vallone et al. [23] found that a campaign that promoted cessation using a positive frames and encouragement was effective for both low and high SES smokers. In a review of the effectiveness of media campaigns across SES groups, Niederdeppe et al. [22] found mixed results: campaigns are sometimes less effective, sometimes equally effective, and sometimes more effective among lower SES populations.

Although studies show that smoking interventions for those with poor mental health can be effective, to our knowledge, no media campaigns have been designed specifically for those with poor mental health. One might expect similarities between the responsiveness of those with poor mental health and those of low SES. Both groups likely experience similar barriers to quitting, including high levels of dependence, high levels of stress, and pro-smoking community or peer group norms that increase social pressure to smoke and exposure to smoking cues [26]. These factors may be even more of a barrier for those with poor mental health. Those with poor mental health have higher levels of negative affect and lower self-efficacy, among other factors that might inhibit cessation and interact with the effectiveness of media messages [27].

In a prior study, Farrelly et al. [16] found that exposure to New York State’s cessation advertising increased smokers’ odds of making a quit attempt, regardless of their level of education or income, and that graphic and/or emotional advertisements were effective and other types of advertisements were not. This paper updates and extends that earlier work by using more recent data and adding two population groups often disproportionately affected by tobacco use: racial/ethnic minorities and those with poor mental health. Our goals for this study are to (1) assess whether exposure to antismoking advertising is associated with making a quit attempt within a number of population subgroups, and (2) determine whether advertisement type differentially affects cessation behavior across subgroups.

## Methods

### Ethics Statement

Protocols were approved by RTI International’s Office of Research Protection Institutional Review Board (Federalwide Assurance #3441) and the Institutional Review Board of the New York State Department of Health.

Survey data were anonymized and de-identified prior to analysis.

### Data

The New York Adult Tobacco Survey (NY-ATS) is a cross-sectional, random-digit-dial telephone survey of adults aged 18 or older in New York State. The survey frame included only landline telephones up to Quarter 3 of 2010 and then included landline and cell phones after that. The NY-ATS includes measures of cigarette and other tobacco product use; smoking cessation; exposure to secondhand smoke; tobacco and cessation-related beliefs, attitudes, and intentions; recall of antismoking advertisements; and sociodemographic characteristics. The NY-ATS has been conducted quarterly from 2003 through 2011, yielding a total sample of 53,706 smokers and nonsmokers. The sample for this study consists of the 9,408 current smokers from the total NY-ATS sample. Data were weighted to reflect the state population of adults, adjusting for different probabilities of selection and survey nonresponse. The New York ATS is sponsored by and available from the New York State Department of Health.

### Measures

#### Quit Attempts

This study is based on a sample of current smokers. Current smoking was defined as having smoked at least 100 cigarettes in one’s lifetime and smoking every day or some days at the time of the interview. The key outcome measure was having made a quit attempt during the past year, measured by asking, “During the past 12 months, have you stopped smoking for 1 day or longer because you were trying to quit smoking?” Response options were yes (1) and no (0).

#### Advertising Exposure

Two measures of advertising exposure were used: potential exposure based on television gross rating points (GRPs) and self-reported confirmed recall of advertisements. Similar to previous studies [28]–[31], the primary measure of interest is the annual number of GRPs for each of the 10 media markets in the state. GRPs are based on the percentage of the population potentially exposed to advertisements (reach) and the average number of times the ads may have been seen (frequency) over a time period. Annual GRPs were calculated by summing the current quarterly GRPs at the time of interview and the three previous quarters. Past-year GRP variables were divided by 5,000 such that an odds ratio (ORs) represents the change in odds for an increase of 5,000 GRPs. Self-reported recall, which has been shown to be highly correlated with media exposure [32], was measured with a series of items that briefly describe each advertisement airing in the quarter. Respondents who indicated recognition were asked for additional details and coded as having confirmed advertisement recall if they provided accurate details. Recall of one or more advertisements for each survey wave was classified as having self-reported recall.

#### Key Sociodemographic Variables

The following sociodemographic variables were included in analyses: age (18–24, 25–39, 40–64, and ≥65 years), gender (female, male), race/ethnicity (non-Hispanic white, non-Hispanic African American, Hispanic), educational attainment (less than high school diploma or equivalent, high school graduate or equivalent, some college, college graduate or more), income (<$30,000; $30,000–$59,999; $60,000–$89,999; ≥$90,000), cigarettes per day (1–9, 10–19, ≥20), and hours of television viewed per day (<3 hours per day, ≥3 hours per day), We also included an indicator variable for those with missing income. Approximately 17% of the pooled sample of smokers had missing income information. Where models were stratified, the corresponding covariate was dropped. Those with missing income were also excluded from the model stratified by income. Mental health was measured by asking, “Now thinking about your mental health, which includes stress, depression, and problems with emotions, for how many days during the past 30 days was your mental health not good?” Poor mental health was defined by responses of “10 or more” days. The specific cut-off used to define poor mental health is somewhat arbitrary, chosen to ensure that at least 10% of the sample would be defined as having “poor mental health”.

To account for potential influences of other tobacco control efforts on outcomes we include an indicator for respondent residing in New York City (versus the rest of the state) and an indicator for increases in the New York State cigarette excise tax (an initial increase in Q2 2008 and then another increase in Q3 2010).

### Coding Advertisement Type

All advertisements measured by the NY-ATS from 2003 through 2011 were coded for strong negative emotions or graphic images. Advertisements that did not include negative emotion or graphic imagery–predominantly those that focus on quitting or exposure to secondhand smoke–were retained for the sake of comparison. Advertisements were reviewed and coded by at least two coders in multiple rounds to resolve differing characterizations. According to Landis and Koch’s [33] k-scale, an indicator of strong negative emotions or graphic images resulted in almost perfect rater agreement (0.81–1.00). Although coders did not code specifically for an efficacy message within each advertisement that might complement the graphic or emotional content [34], all advertisements informed smokers about the state’s quitline. Coders also classified advertisements by type (e.g., cessation, dangers of secondhand smoke). Only cessation-related advertisements were examined in the analyses.

### Analyses

We estimated descriptive statistics for all key variables used in our models averaged across the pooled sample of NY-ATS smokers for 2003 through 2011. We also estimated descriptive statistics for quit attempts in the past 12 months, confirmed awareness of advertisements, and hours of weekly television viewing for each subgroup. To address the primary purpose of this paper, two levels of analysis were conducted. The first examined the effect of New York State’s antismoking advertising overall on making a quit attempt in the past 12 months. The second explored whether past 12-month quit attempts were related specifically to advertisements with graphic imagery or strong negative emotion. Each level of analysis was conducted with both advertising exposure measures (GRPs and confirmed advertising recall). To address the first level of analysis, quit attempts were regressed on advertising exposure, controlling for the sociodemographic variables noted above. To address the second level of analysis, quit attempts were regressed on advertising exposure for graphic and/or emotional content and comparison advertisements separately, controlling for sociodemographic variables.

Regressions were stratified by race/ethnicity, education (less than a high school degree vs. higher education levels), income (<$30,000 vs. ≥$30,000), and mental health (poor vs. not poor mental health). All regressions were weighted and accounted for the survey design using Stata, version 12, in 2012. GRP information was available for the full study period (Quarter 3 of 2003 through Quarter 4 of 2011), whereas complete confirmed recall data were available only from Quarter 2 of 2004 through Quarter 4 of 2011. Thus, GRP models include between 7,899 and 8,916 smokers depending on the stratifying variable of the model, and confirmed recall models include between 6,926 and 7,945 smokers.

## Results

Weighting of the sample provided for sociodemographic proportions in line with current population estimates (Table 1). Mean daily television viewing for the sample overall was 3.2 hours. Mean television consumption by sociodemographic subgroups varied: white (non-Hispanic) and other respondents viewed an average of 2.9 hours of television daily, whereas black (non-Hispanic) and Hispanic respondents viewed an average of 4.2 and 3.4 hours of television per day, respectively (Table 2). This generally corresponded with levels of advertisement awareness–black (non-Hispanic) and Hispanic respondents had the highest levels of confirmed awareness in the most recent 3 years of survey data (2009–2011): 38.6% and 35.2%, respectively. Additionally, television viewing was higher among respondents with less education, lower income, and poor mental health.

**Table 1:** Sample Descriptive Statistics, Pooled Average, New York Adult Tobacco Survey, 2003–2011.

Variable	%	N
**Age**		
18–24	12.4%	2,854
25–39	25.9%	9,978
40–64	44.0%	26,606
≥65	17.6%	13,589
**Gender**		
Female	52.5%	33,331
Male	47.5%	20,374
**Race/ethnicity**		
White (non-Hispanic)	69.1%	43,523
Black (non-Hispanic)	15.3%	5,913
Hispanic	15.6%	4,270
**Education**		
Less than high school diploma	8.3%	3,894
High school graduate	27.2%	14,806
Some college	25.7%	13,675
College graduate	38.9%	21,112
**Income (annual)**		
<$30,000	23.6%	14,096
$30,000–$59,999	24.3%	14,134
$60,000–$89,999	14.5%	7,655
≥$90,000	20.3%	9,058
Missing	17.3%	8,763
**Mental health**		
Good mental health	88.2%	46,562
Poor mental health	11.8%	6,229
**Cigarettes smoked per day**		
<9	38.5%	3,074
10 to 19	29.1%	2,671
≥20	32.4%	3,446
**Weekly television viewing**		
Mean (hours)	3.2	52,950
<3 hours per week	52.0%	27,550
≥3 hours per week	48.0%	25,400
**Quit attempt in past 12 months**		
No	45.8%	4,293
Yes	54.1%	5,072

**Table 2:** Awareness, Media Consumption by Subgroups.

Subgroup	Made Quit Attempt in Past Year (most recent 3 years)	Awareness (most recent 3 years)	Daily Television Viewing (Mean Hours)
**Race/ethnicity**			
White (non-Hispanic)	50.9% (56.3%)	21.5% (29.7%)	2.9
Black (non-Hispanic)	61.6% (68.3%)	27.8% (38.6%)	4.2
Hispanic	60.7% (60.3%)	26.8% (35.2%)	3.4
**Education**			
Less than high school diploma	54.7% (63.0%)	23.7% (32.9%)	3.9
Some college or more	53.4% (55.1%)	23.1% (31.5%)	2.8
**Income (annual)**			
<$30,000	57.1% (62.6%)	24.4% (33.1%)	4.0
≥$30,000	52.2% (55.9%)	23.7% (32.6%)	2.8
**Mental health**			
Good mental health	53.1% (58.9%)	23.2% (32.0%)	3.1
Poor mental health	57.7% (59.3%)	24.5% (33.4%)	4.1

### The Effect of Antismoking Advertising on Quit Attempts

#### Race/Ethnicity

Greater GRP level was associated with a higher likelihood of making a quit attempt among white non-Hispanic respondents (OR 1.28; *p*<0.001) and Hispanic respondents (OR 1.41; *p*<0.05) (Table 3). Confirmed advertisement recall was associated with a higher likelihood of making a quit attempt among white non-Hispanic respondents (OR 1.38; *p*<0.01) and black non-Hispanic respondents (OR 1.99; *p*<0.01).

**Table 3:** Advertising Exposure’s Effect on Quit Attempts by Race/Ethnicity, New York Adult Tobacco Survey, 2003–2011.

	White, non-Hispanic		Black, non-Hispanic		Hispanic	
	OR		OR		OR	
Variable	[95% CI]	N	[95% CI]	N	[95% CI]	N
**Past year GRPs**	1.28***	7,038	1.19	1,149	1.41*	729
	[1.11,1.48]		[0.91,1.55]		[1.01,1.97]	
**Past year GRPs by ad type**		7,038		1,149		729
Graphic and/or emotional advertisements	1.44***		1.35		1.51	
	[1.18,1.75]		[0.93,1.97]		[0.97,2.34]	
Comparison advertisements	1.08		0.91		1.19	
	[0.89,1.32]		[0.54,1.53]		[0.56,2.51]	
**Confirmed recall**	1.38**	6,279	1.99**	1,015	1.17	651
	[1.11,1.72]		[1.28,3.09]		[0.65,2.11]	
**Confirmed recall of graphic and or/emotional ads**	1.49***	6,170	2.00**	997	1.21	646
	[1.18,1.88]		[1.25,3.19]		[0.66,2.21]	

*p<0.05,

**p<0.01,

***p<0.001.

Note: Regressions that use confirmed awareness as the key covariate cover the period April 2004–2011, whereas regressions that use gross rating points (GRPs) as the key covariate cover the period 2003–2011. Regressions controlled for age (18–24, 25–39, 40–64, ≥65), gender (male, female), education (less than high school diploma, high school diploma or GED, some college, college graduate), annual income (<$30,000; $30,000–$59,999; $60,000–$89,999; ≥$90,000; missing income), cigarettes smoked per day (<10, 10 to 19, ≥20), daily television viewing (<3 hours, ≥3 hours), respondent residence (New York City, Rest of New York state), and increases in state cigarette taxes (pre-, post-).

#### Education

Greater GRP level and confirmed advertisement recall were associated with a higher likelihood of making a quit attempt among respondents regardless of their level of educational attainment: an odds ratio of 1.24 (*p*<0.05) among those with a high school diploma or less and an odds ratio of 1.33 (*p*<0.001) among those with some college education or more (Table 4). Using the confirmed advertisement recall measure, the odds ratio was 1.44 (*p*<0.01) for those with a high school diploma or less and 1.35 (*p*<0.05) among those with more education.

**Table 4:** Advertising Exposure’s Effect on Quit Attempts by Education, New York Adult Tobacco Survey, 2003–2011.

	High School Graduate or Less		Some College or More	
	OR		OR	
Variable	[95% CI]	N	[95% CI]	N
**Past year GRPs**	1.24*	4,248	1.33***	4,668
	[1.04,1.49]		[1.13,1.56]	
**Past year GRPs by ad type**		4,248		4,668
Graphic and/or emotional advertisements	1.47**		1.42**	
	[1.15,1.88]		[1.15,1.76]	
Comparison advertisements	0.96		1.17	
	[0.73,1.25]		[0.91,1.50]	
**Confirmed recall**	1.44**	3,784	1.35*	4,161
	[1.10,1.89]		[1.04,1.76]	
**Confirmed recall of graphic and or/emotional ads**	1.61**	3,722	1.34*	4,091
	[1.21,2.15]		[1.01,1.77]	

*p<0.05,

**p<0.01,

***p<0.001.

Note: Regressions that use confirmed awareness as the key covariate cover the period April 2004–2011, whereas regressions that use gross rating points (GRPs) as the key covariate cover the period 2003–2011. Regressions controlled for age (18–24, 25–39, 40–64, ≥65), annual income (<$30,000; $30,000–$59,999; $60,000–$89,999; ≥$90,000; missing income), gender (male; female), cigarettes smoked per day (<10, 10 to 19, ≥20), daily television viewing (<3 hours, ≥3 hours), respondent residence (New York City, Rest of New York state), and increases in state cigarette taxes (pre-, post-).

#### Income

Greater GRP level was associated with a higher likelihood of making a quit attempt among respondents regardless of their income: the odds ratio was 1.39 (*p*<0.01) among those earning less than $30,000 per year and 1.24 (*p*<0.01) among those earning more (Table 5). Confirmed advertisement recall was associated with a higher likelihood of making a quit attempt for those earning less than $30,000 (OR 1.52; *p*<0.01) and those earning $30,000 or more (OR 1.46; *p*<0.01).

**Table 5:** Advertising Exposure’s Effect on Quit Attempts by Annual Income, New York Adult Tobacco Survey, 2003–2011.

	<$30,000		≥$30,000	
	OR		OR	
Variable	[95% CI]	N	[95% CI]	N
**Past year GRPs**	1.39**	3,352	1.24**	4,547
	[1.11,1.73]		[1.06,1.46]	
**Past year GRPs by ad type**		3,352		4,547
Graphic and/or emotional advertisements	1.62**		1.38**	
	[1.18,2.21]		[1.12,1.70]	
Comparison advertisements	1.11		1.03	
	[0.82,1.51]		[0.80,1.32]	
**Confirmed recall**	1.52**	3,011	1.46**	4,032
	[1.11,2.08]		[1.12,1.91]	
**Confirmed recall of graphic and or/emotional ads**	1.73**	2,965	1.47**	3,961
	[1.24,2.42]		[1.11,1.96]	

*p<0.05,

**p<0.01,

***p<0.001.

Note: Regressions that use confirmed awareness as the key covariate cover the period April 2004–2011, whereas regressions that use gross rating points (GRPs) as the key covariate cover the period 2003–2011. Regressions controlled for age (18–24, 25–39, 40–64, ≥65), gender (male; female), education (less than high school diploma, high school diploma or GED, some college, college graduate), cigarettes smoked per day (<10, 10 to 19, ≥20), daily television viewing (<3 hours, ≥3 hours), respondent residence (New York City, Rest of New York state), and increases in state cigarette taxes (pre-, post-).

#### Mental Health

Greater GRP level (OR 1.33; *p*<0.001) and confirmed advertisement recall (OR 1.46; *p*<0.001) were associated with a higher likelihood of making a quit attempt among respondents with good mental health, but not among those with poor mental health (Table 6).

**Table 6:** Advertising Exposure’s Effect on Quit Attempts by Mental Health Status, New York Adult Tobacco Survey, 2003–2011.

	Poor Mental Health		Good Mental Health	
	OR		OR	
Variable	[95% CI]	N	[95% CI]	N
**Past year GRPs**	1.13	2,019	1.33***	6,758
	[0.87,1.46]		[1.16,1.53]	
**Past year GRPs by ad type**		2,019		6,758
Graphic and/or emotional advertisements	1.27		1.49***	
	[0.89,1.82]		[1.24,1.79]	
Comparison advertisements	0.9		1.1	
	[0.62,1.30]		[0.89,1.36]	
**Confirmed recall**	1.07	1,800	1.46***	6,021
	[0.75,1.53]		[1.17,1.82]	
**Confirmed recall of graphic and or/emotional ads**	1.12	1,772	1.54***	5,919
	[0.77,1.64]		[1.21,1.95]	

*p<0.05,

**p<0.01,

***p<0.001.

Note: Regressions that use confirmed awareness as the key covariate cover the period April 2004–2011, whereas regressions that use gross rating points (GRPs) as the key covariate cover the period 2003–2011. Regressions controlled for age (18–24, 25–39, 40–64, ≥65), gender (male; female), education (less than high school diploma; high school diploma or GED; some college; college graduate), annual income (<$30,000; $30,000–$59,999; $60,000–$89,999; ≥$90,000; missing income), cigarettes smoked per day (<10, 10 to 19, ≥20), daily television viewing (<3 hours, ≥3 hours), respondent residence (New York City, Rest of New York state), and increases in state cigarette taxes (pre-, post-).

### The Effect of Advertisement Type on Quit Attempts

#### Race/Ethnicity

Greater graphic/negative advertising GRP level was associated with an increased likelihood of making a quit attempt among white non-Hispanic (OR 1.44; *p*<0.001) (see Table 3). Comparison group advertising GRP level was not significantly associated with quit attempts in any of the racial/ethnic subgroups.

Black non-Hispanic respondents who had confirmed recall of graphic and/or emotional advertising were more likely to make a quit attempt than their counterparts who could not recall any advertising or were aware of only comparison advertising (OR 2.00; *p*<0.01). White non-Hispanic respondents who had confirmed recall of graphic/negative advertising were more likely to make a quit attempt than those who were unaware of any advertising or aware of comparison advertising only (OR 1.49; *p*<0.001).

#### Education

Greater graphic/negative advertising GRP level was associated with an increased likelihood of making a quit attempt in both education groups: the odds ratio was 1.47 (*p*<0. 01) among those with a high school diploma or less and 1.42 (*p*<0.01) among those with some college education or more (see Table 4).

Respondents with a high school diploma or less who had confirmed recall of graphic and/or emotional advertising were more likely to make a quit attempt than their counterparts who could not recall any advertising or were aware of only comparison advertising (OR 1.61; *p*<0.01). Likewise, respondents with some college education or more who had confirmed recall of graphic and/or emotional advertising were more likely to make a quit attempt than their counterparts who could not recall any advertising or were aware of only comparison advertising (OR 1.34; *p*<0.05).

#### Income

Greater graphic/negative advertising GRP level was associated with an increased likelihood of making a quit attempt in both income categories: the odds ratio was 1.62 (*p*<0.01) among those whose income was less than $30,000 per year and 1.38 (*p*<0.01) among those whose income was greater (see Table 5).

Respondents whose income was less than $30,000 per year who had confirmed recall of graphic and/or emotional advertising were more likely to make a quit attempt than their counterparts who could not recall any advertising or were only aware of comparison advertising (OR 1.73; *p*<0.01). Similarly, those making $30,000 per year or more who were aware of graphic and/or emotional advertising were more likely to make a quit attempt that their counterparts who could not recall any advertising or were only aware of comparison advertising (OR 1.47; *p*<0.01).

#### Mental Health

Greater graphic/negative advertising GRP level was associated with an increased likelihood of making a quit attempt among those with good mental health (OR 1.49; *p*<0.001) (see Table 6). Respondents with good mental health who had confirmed recall of graphic and/or emotional advertising were more likely to make a quit attempt than their counterparts who could not recall any advertising or were aware of comparison advertising only (OR 1.54; *p*<0.001). Advertising exposure was not significantly associated with quit attempts among respondents with poor mental health.

## Discussion

This study yields three important findings. First, antismoking advertising promotes quit attempts among racial/ethnic minority smokers and smokers of lower education and income. Second, advertising effectiveness is attributable in part to advertisements with graphic imagery or strong negative emotion. Third, smokers with poor mental health do not appear to benefit from exposure to antismoking advertising of any type.

The finding of this study–that general population antismoking advertising can be used to promote quit attempts within several particularly vulnerable populations–represents a contribution to the scant literature on the effectiveness of media campaigns by race/ethnicity and SES. This finding suggests that a single comprehensive campaign intended for all smokers would be sufficient to influence the cessation behavior of racial/ethnic minority populations and individuals of lower education or income. Campaign planners would not necessarily need to develop and deliver separate campaigns for each vulnerable population. It should be noted that the effect of advertisements appears weaker for Hispanics in our sample. This result does not appear to be driven by differences in media consumption since Hispanics watch a similar amount of television as other groups in our data. Perhaps this could be due to differences in advertisement exposure on Spanish television or differences in the perceived effectiveness of the advertisements (in English or Spanish) by Hispanics.

The second finding of this study is that the effectiveness of the New York State antismoking advertising is attributable, at least in part, to the graphic imagery or strong negative emotion that characterizes many of the advertisements. This study is not the first to suggest that negative emotional advertising resonates with smokers and produces greater behavior change than advertisements of other types (see, for example, Durkin et al. [20] for a study of the media campaign in Massachusetts). Nevertheless, the evidence in this area is only recently beginning to emerge. Future research should continue to document the effectiveness of various advertisement types, including whether quit attempts associated with negative emotional advertising translate into long-term successful quitting and whether the genre “graphic imagery and/or negative emotion imagery” can be further refined.

This study is perhaps the first to examine the effects of a cessation media campaign on smokers with self-reported poor mental health. The finding that those with poor mental health do not benefit from exposure to antismoking advertising potentially points to a concern for campaign planners and those interested in lowering smoking among those with poor mental health. This study suggests the possibility that past general population campaigns–which were expected to serve all smokers–have not, in fact, served those with mental illness. This finding does not appear to be driven by lower levels of media consumption among those with poor mental health; our data show that those with poor mental health watched more hours of television per week than those with good mental health. It may suggest that graphic and/or negative advertisements do not resonate with or otherwise prompt behavior change in this population. Perhaps a stronger efficacy or motivational message would be more effective. Future research is needed to understand what type of media campaign might effectively increase cessation activity in this population.

The study has several limitations. First, the analysis relies on a repeated cross-sectional design. A longitudinal study would provide stronger conclusions about the effectiveness of advertising. Second, although the study makes use of variation in exposure to advertising across 10 media markets over a 7-year period, the results are based on the experience of a single state, limiting our ability to easily account for concomitant tobacco control activities or rule out alternative explanations. However, the similarity of results using self-reported recall and advertising dose suggests that the current results are robust. Third, our measure of mental health status is crude, which may result in misclassification of mental health status. We did compare the smoking prevalence of those classified as having poor mental health to those with good mental health, and, as expected, those with poor mental health had significantly higher smoking prevalence than those with good mental health (33% vs. 14%), Future studies should examine the relationship between exposure to antismoking advertisements and smoking among those with poor mental health using reliable and validated measures of mental health. In addition, our sample likely also does not include those with more serious mental illness, and thus our results may not generalize to the general population of those suffering from mental illness.

This study contributes to the evidence about how cessation media campaigns can be used most effectively to increase quit attempts within vulnerable subgroups. In particular, it suggests that a general campaign can impact cessation among a range of sociodemographic groups. Further, this study points to the need for more research to understand what message strategies might work for those with poor mental health.
